# Cervicogenic headache in forward head posture: frequency and associated factors in a cross-sectional study

**DOI:** 10.22514/jofph.2025.061

**Published:** 2025-09-12

**Authors:** Ahmet Usen, Merve Demiroz Gunduz

**Affiliations:** ^1^Department of Physical Medicine and Rehabilitation, Faculty of Medicine, Istanbul Medipol University, 34214 Istanbul, Türkiye

**Keywords:** Cervicogenic headache, Head, Posture, Cervical vertebrae

## Abstract

**Background**: This study aimed to determine the frequency and associated factors of cervicogenic headache (CGH) in individuals with forward head posture (FHP). Additionally, craniovertebral angle (CVA)-related factors were examined in patients diagnosed with CGH. **Methods**: This cross-sectional study included 117 patients aged 18–45 years who presented with neck pain and were identified with FHP. CGH diagnosis was based on the International Classification of Headache Disorders (ICHD-3) criteria. CVA was measured using posture analysis software, and assessments included the Neck Disability Index (NDI), Henry Ford Headache Disability Inventory (HDI-T), Headache Impact Scale (HIT-6), Pittsburgh Sleep Quality Index (PSQI), Beck Depression Inventory (BDI), and Visual Analog Scale (VAS) for pain. Statistical analyses included independent *t*-tests, chi-square tests and logistic regression models. **Results**: The frequency of CGH in patients with FHP was 53.8%. Compared to the non-CGH group, those with CGH had significantly lower CVA (*p* = 0.030) and higher PSQI (*p* = 0.001) and BDI scores (*p* < 0.001). Logistic regression analysis identified low CVA (Odds Ratio (OR): 0.878, *p* = 0.014) and poor sleep quality (OR: 1.140, *p* = 0.025) as independent predictors of CGH. Additionally, Body Mass Index and VAS scores were negatively correlated with CVA (*p* < 0.05). **Conclusions**: FHP may be associated with CGH, possibly through increased biomechanical load and neuromechanical sensitivity. Interventions such as corrective exercises, weight management, and improving sleep quality may be considered as supportive strategies in CGH management; however, causal relationships cannot be inferred from this study. Further studies are needed to explore the long-term effects of postural interventions on CGH.

## 1. Introduction

Cervicogenic headache (CGH) is a type of secondary headache that originates in the cervical spine and is felt in the head [1]. It is usually defined as a unilateral headache triggered by neck movements and neck-related symptoms. Its basic mechanism is based on the neural convergence between the trigeminal nerve and the upper three cervical nerves (C1, C2 and C3) [2]. This process is also defined as trigeminocervical convergence at the trigeminal nucleus caudalis level [3]. This leads to the perception of pain signals from the neck as headaches. The source of pain is often thought to be structures such as upper cervical joints, muscles, ligaments, intervertebral discs, and veins. However, current perspectives suggest that muscular sources may be more characteristic of tension-type headache, while upper cervical facet joints are increasingly emphasized in CGH [4]. Dysfunction in these anatomical structures can generate pains that are reflected in the head through the complex nerve network of the cervical spine [2]. Understanding the mechanism of CGH is of great importance for both diagnosis and treatment. While the one-year prevalence in the adult population ranges from 0.2% to 2.2% [5, 6], this rate increases to 10.4% in the young population and has been associated with sustained neck loading during prolonged use of digital devices such as smartphones and computers [7].

Forward head posture (FHP) may predispose individuals to the development of CGH by increasing the biomechanical load on the cervical spine [8]. The increasing use of information and communication technologies such as mobile phones and computers causes individuals to keep their necks tilted forward for long periods of time and contributes to the formation of FHP [9]. This posture, which is characterized by a decrease in the craniovertebral angle (CVA) and the forward placement of the head in the sagittal plane relative to the shoulders, leads to a deterioration of the balance between the cervical muscles and an increase in mechanosensitivity [10]. In the literature, it has been shown that FHP is associated with restriction of cervical flexion movement, increased neck pain severity, and postural balance disorders [11]. Additionally, FHP involves upper cervical extension, leading to shortening and increased activation of the rectus capitis posterior major and minor muscles. These changes may contribute to nociceptive input from the upper cervical joints, a mechanism implicated in cervicogenic headaches [12]. Furthermore, this posture is reported to trigger chronicity of CGH through shortening of the suboccipital muscles, tension in the trapezius muscle, and structural changes [13]. In addition, it has been stated that the negative effects of FHP on neck pain and loss of functionality increase pain severity, especially in adults, and adversely affect musculoskeletal health [14]. Such findings suggest the need for extensive research to understand the relationship between FHP and CGH better and to develop effective approaches to the management of neck pain. Although previous studies have investigated the relationship between FHP and CGH using postural or clinical measures, their findings were limited by methodological inconsistencies and a lack of integration between postural, clinical, and psychosocial variables [15, 16].

The aim of this study was to determine the frequency and associated factors of CGH in patients who presented to our outpatient clinic with neck pain and were identified as having FHP. In particular, the study explored the prevalence of CGH in this population, the demographic and clinical features associated with its occurrence, and whether specific CVA-related characteristics could differentiate patients with CGH from those without. The findings are expected to contribute new insights into the mechanisms underlying CGH in the context of postural disorders and improve the clinical management of patients presenting with FHP-related neck pain. We hypothesized that CVA-related postural parameters, sleep quality, and BMI would be significantly associated with the presence of CGH in this population.

## 2. Materials and methods

### 2.1 Study design and participants

This was a cross-sectional study conducted in the Department of Physical Medicine and Rehabilitation, Medipol Mega Hospitals Complex. Data collection was carried out between July 2024 and January 2025. The sample size was determined via power analysis with 80% power, 5% significance level, and an effect size of 0.30. As a result of the analysis, it was calculated that at least 105 participants should be included in order to provide reliable and valid results. Considering a dropout rate of 10%, a total of 117 participants were required.

All participants presented directly to the physical medicine and rehabilitation outpatient clinic without prior referral by a neurologist or general practitioner. Patients who applied to the outpatient clinic with neck pain and had a CVA measurement of ≤50° were included in this study. Patients with spinal deformities such as cervical vertebral fractures, scoliosis, or kyphosis, patients with vertigo, osteoporosis, tumors or inflammatory diseases affecting the cervical region, and patients with known symptoms of vertebrobasilar insufficiency or cervical spine instability were excluded from the study.

### 2.2 Data collection

The following assessment tools were applied to each participant.

#### 2.2.1 Neck disability index (NDI)

The NDI is a scale that evaluates the impact of neck pain on daily life. The Turkish validity and reliability study was conducted by Aslan *et al*. [17] (Intraclass Correlation Coefficient (ICC) = 0.979). It consists of a total of 10 questions and is scored between 0–100, with higher scores meaning more disability.

#### 2.2.2 Henry Ford headache disability inventory (HDI-T)

HDI-T, a 21-item scale with a two-dimensional structure, emotional and functional, is used to measure headache disability. It is scored on a scale of 0–100, with high scores indicating more disability. The Turkish validity and reliability study was conducted by Kılınç *et al*. [18] (ICC = 0.901). Unlike its original form, it contains 21 items, not 25.

#### 2.2.3 Headache impact scale (HIT-6)

This is a 6-item scale that evaluates the impact of headache on social and physical functioning. The Turkish validity and reliability study was conducted by Dikmen *et al*. [19] (Cronbach’s α = 0.753–0.864; *r* = 0.437). Each question is graded between 6 and 13 points, and the total score ranges from 36 to 78. As the score increases, the severity of the headache’s impact on an individual’s life also increases.

#### 2.2.4 Pittsburgh sleep quality index (PSQI)

This is a self-report scale that evaluates sleep quality and disorders over the past month. The Turkish validity and reliability study was conducted by Ağargün *et al*. [20] (Cronbach’s α = 0.80; *r* = 0.98). It consists of a total of 24 questions; 19 are self-reported, and five are answered by a spouse or roommate. The PSQI total score ranges from 0 to 21, and sleep quality is considered to worsen as the score increases.

#### 2.2.5 Beck depression inventory (BDI)

This scale evaluates the emotional, cognitive, somatic, and motivational components of depression. The Turkish validity and reliability study was conducted by Hisli [21] (Cronbach’s α = 0.80). The scale consists of 21 items and is scored on a scale of 0–63. High scores indicate an increase in the severity of depression.

Demographic (age, sex, height, weight, smoking, alcohol consumption) and clinical information including headache duration (per year), frequency (how many days in the past week and how many hours in total), and severity (evaluated with the Visual Analog Scale (VAS)) were also collected from participants.

### 2.3 Evaluation and measurements

#### 2.3.1 Craniovertebral angle (CVA)

CVA was measured by evaluating photographs taken with a reflex camera (Nikon Model D5300 SLR, Tokyo, Japan) using posture analysis software [22]. The camera was fixed at a distance of 3 meters from the standing participant, and the participant was asked to take a comfortable posture. The craniovertebral angle (α) is defined as the angle formed between a horizontal line drawn parallel to the ground at the level of the spinous process of C7 and an oblique line extending from the tragus of the ear to the spinous process of C7 (Fig. 1). Measurements were reported to provide high reliability with an ICC value of 0.98 and good validity compared to radiography (*r* = 0.89) [23].

**Fig. 1.** The measurement of craniovertebral angle (α) defined as the angle formed between a horizontal line drawn parallel to the ground at the level of the spinous process of the seventh cervical vertebra (C7) and an oblique line extending from the tragus of the ear to the spinous process of C7.

#### 2.3.2 Cervical joint range of motion (ROM)

Flexion, extension, right lateral flexion, left lateral flexion, right rotation and left rotation movements were measured using a goniometer.

#### 2.3.3 Diagnosis of cervicogenic headache

Participants’ headaches were evaluated according to the criteria of the International Classification of Headache Disorders (ICHD-3) [1]. CGH was diagnosed based on the following criteria: (1) temporal onset of headache in relation to a cervical disorder or trauma, (2) headache triggered by cervical movement or external pressure, (3) ipsilateral neck pain accompanying the headache, and (4) cervical pathology confirmed by imaging. All assessments were conducted by a physiatrist with clinical experience in headache management. It is important to note that patients with overlapping headache types (*e.g.*, coexisting migraine or tension-type headache) were not excluded, as CGH may coexist with other headache disorders in real-world clinical settings.

### 2.4 Statistical analysis

The data obtained in this study were analyzed using IBM SPSS Statistics software (Version 26.0, IBM Corp., Armonk, NY, USA). The normality distribution of the data was evaluated by the Kolmogorov-Smirnov test. Continuous variables are expressed as mean ± standard deviation because they meet the assumption of normality. Independent sample *t*-test was used to compare the groups. Categorical variables were summarized as frequency (n) and percentage (%). The Chi-square test or, where appropriate, Fisher’s Exact Test was used to compare categorical variables.

The relationships between clinical and functional parameters (*e.g.*, VAS, headache frequency and duration, CVA, ROM, PSQI, BDI, NDI, HDI-T, HIT-6) were analyzed using the Pearson correlation coefficient. Logistic regression analysis was performed to evaluate the effects of independent variables (CVA, PSQI, BDI, BMI and age) on the dependent variable (presence of CGH). The goodness-of-fit of each logistic regression model was evaluated using the Hosmer-Lemeshow test. For the univariable models constructed with age (73.5% accuracy, 77.8% sensitivity, 68.5% specificity; χ^2^(7) = 36.124, *p* < 0.001), CVA (65.8% accuracy, 81.0% sensitivity, 48.1% specificity; χ^2^(7) = 40.893, *p* < 0.001), and BDI (75.2% accuracy, 81.0% sensitivity, 68.5% specificity; χ^2^(8) = 32.983, *p* < 0.001), the Hosmer-Lemeshow test indicated poor model fit due to statistically significant results. In contrast, the model based on PSQI (64.1% accuracy, 69.8% sensitivity, 57.4% specificity; χ^2^(8) = 12.842, *p* = 0.117) and the multivariable model including age, CVA and PSQI (65.0% accuracy, 81.0% sensitivity, 46.3% specificity; χ^2^(8) = 7.802, *p* = 0.453) did not show significant lack of fit, indicating an acceptable alignment between observed and predicted values. The significance level was accepted as *p* < 0.05 with Odds Ratio (OR) and 95% Confidence Intervals (CI).

## 3. Results

Table 1 compares the demographic and sociocultural data of the CGH and non-CGH groups. In the analysis, there was no statistically significant difference between the two groups in terms of BMI, sex, smoking status and alcohol consumption (all *p* > 0.05). However, the mean age was higher in the CGH group than in the non-CGH group (33.25 ± 5.56 years *vs.* 28.54 ± 8.35 years, *p* < 0.001).

**Table 1.** Distribution of demographic data by CGH and non-CGH groups.

Variables	Groups	non-CGH Group	CGH Group	*p*
Sex, n (%)				
	Female	24 (40.7)	35 (59.3)	0.231
	Male	30 (51.7)	28 (48.3)	
Smoking, n (%)				
	Yes	15 (51.7)	14 (48.3)	0.488
	No	39 (44.3)	49 (55.7)	
Alcohol, n (%)				
	Yes	8 (53.3)	7 (46.7)	0.550
	No	46 (45.1)	56 (54.9)	
Age (yr) (Mean ± SD)		28.54 ± 8.35	33.25 ± 5.56	<0.001*
BMI (Mean ± SD)		24.26 ± 4.28	23.68 ± 3.09	0.899

CGH: Cervicogenic Headache; BMI: Body Mass Index (kg/m^2^); SD: Standard Deviation; p: p-value. *: p < 0.05 refers to the level of statistical significance.

Table 2 compares the clinical and functional data of CGH and non-CGH groups. CVA value was significantly lower in the CGH group compared to the non-CGH group (38.95 ± 4.65 *vs.* 41.11 ± 5.97, *p* = 0.030). PSQI (6.96 ± 4.18 *vs.* 9.48 ± 3.92, *p* = 0.001) and BDI scores (9.74 ± 7.37 *vs.* 14.79 ± 6.48, *p* < 0.001) were significantly higher in the CGH group than in the non-CGH group. Notably, no significant difference was observed between the two groups in terms of cervical joint range of motion (flexion, extension, lateral and rotation movements) and NDI scores (*p* > 0.05).

**Table 2.** Comparison of clinical and functional data by CGH and non-CGH groups.

Variables	non-CGH Group	CGH Group	*t*	*p*
CVA	41.11 ± 5.97	38.95 ± 4.65	2.196	0.030*
FLEX	57.98 ± 3.40	57.19 ± 4.46	1.064	0.289
EXT	65.39 ± 4.55	65.51 ± 5.43	−0.127	0.899
R LAT	37.72 ± 3.59	37.02 ± 2.77	1.200	0.232
L LAT	38.78 ± 3.20	38.81 ± 3.12	−0.054	0.957
R ROT	70.11 ± 5.45	68.30 ± 6.23	1.658	0.100
L ROT	69.52 ± 5.52	67.73 ± 6.26	1.625	0.107
NDI	32.63 ± 10.94	35.49 ± 9.55	−1.511	0.134
PSQI	6.96 ± 4.18	9.48 ± 3.92	−3.352	0.001*
BDI	9.74 ± 7.37	14.79 ± 6.48	−3.945	<0.001*
VAS		6.30 ± 2.17	−21.351	N/A
Headache Duration (yr)		7.70 ± 4.55	−12.417	N/A
Headache Days (Last wk)		2.46 ± 1.53	−11.788	N/A
Headache Hours (Last wk)		17.57 ± 19.86	−6.496	N/A
HDI-T		35.60 ± 16.03	−16.311	N/A
HIT		62.29 ± 5.09	−37.942	N/A

CGH: Cervicogenic Headache; CVA: Craniovertebral Angle; FLEX: Cervical Flexion (degrees); EXT: Cervical Extension (degrees); R LAT: Right Lateral Flexion (degrees); L LAT: Left Lateral Flexion (degrees); R ROT: Right Rotation (degrees); L ROT: Left Rotation (degrees); NDI: Neck Disability Index; PSQI: Pittsburgh Sleep Quality Index; BDI: Beck Depression Inventory; VAS: Visual Analog Scale; HDI-T: Henry Ford Headache Disability Inventory; HIT: Headache Impact Test; N/A: Not applicable. *: Refers to the statistical significance level as p < 0.05.

According to the results of the univariable logistic regression analysis, age (OR = 1.10, 95% CI: 1.04–1.16, *p* = 0.001), CVA (OR = 0.926, 95% CI: 0.863–0.994, *p* = 0.032), PSQI (OR = 1.167, 95% CI: 1.059–1.287, *p* = 0.002) and BDI (OR = 1.115, 95% CI: 1.049–1.184, *p* < 0.001) showed significant relationships with CGH (Table 3). In multivariable logistic regression analysis, CVA (OR = 0.892, 95% CI: 0.812–0.979, *p* = 0.017) was determined as an independent predictor for CGH (Table 3). When PSQI was added to the multivariable model, it was determined that PSQI (OR = 1.140, 95% CI: 1.017–1.279, *p* = 0.025) was an independent predictor for CGH in addition to CVA (OR = 0.878, 95% CI: 0.792–0.974, *p* = 0.014) (Table 3).

**Table 3.** Factors associated with cervicogenic headache: univariable and multivariable logistic regression analysis results.

Variables	Univariable analysis			Multivariable Model 1			Multivariable Model 2		
	OR	95% CI	*p*	OR	95% CI	*p*	OR	95% CI	*p*
Age (yr)	1.101	1.041–1.165	0.001*	1.062	0.995–1.134	0.072	1.052	0.982–1.128	0.151
CVA	0.926	0.863–0.994	0.032*	0.892	0.812–0.979	0.017*	0.878	0.792–0.974	0.014*
PSQI	1.167	1.059–1.287	0.002*				1.140	1.017–1.279	0.025*
BDI	1.115	1.049–1.184	<0.001*						

OR: Odds Ratio; CI: Confidence Interval; p: p-value; CVA: Craniovertebral Angle; PSQI: Pittsburgh Sleep Quality Index; BDI: Beck Depression Inventory. *: p < 0.05 refers to the statistical significance level. Model 1: Multivariable logistic regression analysis including Age and CVA. Model 2: Multivariable logistic regression analysis including Age, CVA and PSQI.

According to the results of the analysis in Table 4, there was a significant negative correlation between CVA and BMI (*r* = −0.449; *p* < 0.001). While there was no significant relationship between CVA and age (*r* = −0.121; *p* = 0.344), a statistically significant negative correlation was found between CVA and VAS (*r* = −0.275; *p* = 0.029). There was no significant relationship between CVA and headache duration (years) and frequency (number of days seen in the last week and total duration) (*p* > 0.05). There was also no statistically significant difference between cervical range of motion and CVA (*p* > 0.05). In addition, a significant positive correlation was found between CVA and PSQI (*r* = 0.323; *p* = 0.010). Although the correlations between CVA and variables such as VAS (*r* = −0.275) and PSQI (*r* = 0.323) were statistically significant, their explanatory power was modest, accounting for approximately 7% and 10% of the variance, respectively. However, there was no statistically significant relationship between CVA and HDI-T, NDI, HIT and BDI (*p* > 0.05).

**Table 4.** Correlation of craniovertebral angle (CVA) with clinical and functional parameters in patients with cervicogenic headache (CGH).

Variables	*r*	*p*
Age (yr)	−0.121	0.344
BMI	−0.449	<0.001*
Headache Duration (yr)	0.055	0.669
Headache Days (Last wk)	0.017	0.897
Headache Hours (Last wk)	0.123	0.339
VAS	−0.275	0.029*
FLEX	0.165	0.195
EXT	0.200	0.117
R LAT	0.099	0.440
L LAT	0.026	0.839
R ROT	0.171	0.181
L ROT	0.199	0.118
HDI-T	0.035	0.785
PSQI	0.323	0.010*
NDI	0.205	0.106
HIT	−0.173	0.176
BDI	0.133	0.299

CVA: Craniovertebral Angle; BMI: Body Mass Index; VAS: Visual Analog Scale; FLEX: Flexion; EXT: Extension; R LAT: Right Lateral Flexion; L LAT: Left Lateral Flexion; R ROT: Right Rotation; L ROT: Left Rotation; HDI-T: Henry Ford Headache Disability Inventory; PSQI: Pittsburgh Sleep Quality Index; NDI: Neck Disability Index; HIT: Headache Impact Test; BDI: Beck Depression Inventory. *: p < 0.05.

According to the results of the multiple linear regression analysis in Table 5, it is seen that the model is significant (*F* = 8.130; *p* < 0.001). In this model, increased BMI (B = −0.645; *p* = 0.001) and increased VAS (B = −0.571; *p* = 0.026) were found to be independent predictors of low CVA. While BMI and VAS were significantly negatively correlated with CVA, PSQI did not have a significant effect on the model (*p* = 0.504).

**Table 5.** Results of multiple linear regression analysis of craniovertebral angle (CVA) with clinical parameters.

Variables	B	SE	*t*	*p*	*F*
BMI	−0.645	0.180	−3.583	0.001*	*F* = 8.130, *p* < 0.001*
VAS	−0.571	0.250	−2.281	0.026*	
PSQI	0.100	0.149	0.672	0.504	

R^2^ (Coefficient of Determination): 0.292. BMI: Body Mass Index (kg/m^2^); VAS: Visual Analog Scale; PSQI: Pittsburgh Sleep Quality Index; B: Unstandardized Regression Coefficient; SE: Standard Error; t: t-statistic; p: p-value; F: F-statistic; *: Refers to the level of statistical significance as p < 0.05.

## 4. Discussion

In this study, we examined the frequency and associated factors of CGH in patients who presented to our outpatient clinic with neck pain and were identified as having FHP. According to our findings, increased age, decreased CVA, and poor sleep quality were significantly associated with CGH. CVA and PSQI were found to be independently associated factors with CGH. However, although both were identified as independent predictors of CGH, the modest odds ratios suggest that other unmeasured variables may also contribute to CGH risk, indicating a multifactorial etiology. In addition, CVA showed a significant negative independent association with BMI and VAS, suggesting the potential impact of these parameters on CGH-related mechanisms.

The prevalence of CGH has been reported with wide variation in previous studies, largely depending on study populations and diagnostic methods. The prevalence of CGH ranges from 0.2% to 64.1%, depending on the type of study population [7, 24, 25]. For example, Kanniappan *et al*. [7] reported that the prevalence of CGH in the young population was 10.4%. On the other hand, Xu *et al*. [24] reported that the prevalence of CGH in individuals over 50 years of age can be up to 42% and suggested that this increase may be due to biomechanical and postural changes depending on age groups. Similarly, in a study conducted among college students, concomitant neck pain was detected in 64.10% of individuals with headaches [25]. Although this may initially suggest a higher prevalence of CGH in the younger population, it should be noted that neck pain is also frequently reported in primary headache disorders such as migraine and tension-type headache due to referred pain mechanisms, which complicates differential diagnosis [26]. In line with the studies mentioned above but with even higher rates, in this study, the frequency of CGH was found to be 53.8% among patients who visited our outpatient clinic for neck pain and were diagnosed with FHP. One of the main reasons for the high prevalence of CGH in our study is that all individuals participating in the study presented with complaints of neck pain. In addition, the evaluation of only patients with FHP may have contributed to this high rate.

Beyond prevalence, the relationship between CGH and cervical alignment, including changes in cervical curvature and postural deviations, has also been widely studied. In the study of Farmer *et al*. [15], which evaluated the relationship between CGH and cervical posture by radiographic analysis, it was stated that an increase in general cervical lordosis increased the likelihood of CGH. In our study, it was found that the CVA value was significantly lower in the CGH group than in the non-CGH group. FHP is known to be associated with decreased lordosis, especially in the lower cervical region (C2–C7). This postural change can increase the biomechanical load on the cervical spine and is associated with greater neck muscle tension and reduced muscle function. Furthermore, FHP can trigger pain mechanisms by causing an imbalance in the cervical musculoskeletal system. Similarly, in the study of Delen and İlter [27], it was reported that the duration of headache was longer in patients with loss of cervical lordosis, but it was not directly related to other pain parameters such as severity and frequency. These findings in the literature support the potential role of changes in cervical lordosis in the development of CGH but do not reveal a definitive causality. In particular, the fact that FHP causes muscle imbalance in the upper cervical segments and increased tension in the suboccipital muscles may be effective in the emergence of CGH by increasing neuromechanical sensitivity.

The biomechanical and physiological mechanisms behind these postural changes are also worth considering. Mechanical stress, postural disorders, and neurophysiological effects play an essential role in the etiology of CGH. In the study conducted by Çoban *et al*. [28], a significant inverse relationship was found between CVA and VAS, and it was shown that CVA is an important parameter in the evaluation of CGH symptoms. Similarly, in our study, a negative correlation was found between low CVA and increased VAS, and these findings are consistent with the results reported in the literature. In this context, Martinez-Merinero *et al*. [29], in their study of 102 patients, reported that the decrease in CVA may increase the mechanosensitivity of tissues through mechanical stress, which may contribute to pain mechanisms in CGH patients. In support of this mechanism, Patwardhan *et al*. [13] suggested that FHP may increase neuromechanical sensitivity in the C2 nerve root by creating shortening of the suboccipital muscles. Furthermore, Chua *et al*. [30] reported that forward head posture exerts continuous pressure on the joint surfaces of the upper cervical vertebrae, which may trigger both peripheral and central sensitization processes. These literature findings support the negative association between low CVA and increased VAS in our study. In addition, Martinez-Merinero *et al*. [29] emphasized that mechanosensitivity is more pronounced in certain regions, especially at the C2 level, reinforcing the importance of CVA in terms of postural and mechanical effects. Moreover, although the CVA difference between CGH and non-CGH groups in our study was approximately 2°, it is notable that Heydari *et al*. [31] reported a minimal clinically important difference (MCID) of 1.40° for CVA, suggesting that this observed change may also have clinical relevance in addition to statistical significance.

In addition to structural and mechanical aspects, psychosocial components such as sleep and depression should also be addressed. In our study, sleep quality was found to be significantly lower, and depression levels were significantly higher in the CGH group. Although impaired sleep quality has also been reported in patients with migraine and tension-type headache [32], observational studies suggest that CGH may involve more specific associations with postural impairments and central sensitization. It has been observed that sleep quality is lower in CGH patients, and the significant relationship between CVA and PSQI suggests a link between postural alignment and sleep quality. Considering the multifactorial nature of CGH, which involves structural, postural, and psychosocial factors, it is also possible that sleep disturbances may emerge as a consequence of persistent cervical discomfort and pain, rather than serving as an initial trigger. Similarly, in the study of Mingels *et al*. [33], which examined the relationships between pain processing, lifestyle, and psychosocial factors in CGH patients, it was shown that sleep quality was significantly worse in the CGH group than in the control group, and this was associated with central sensitivity symptoms. In another study by Mingels *et al*. [34], the relationship between individual differences in mechanical pain sensation and biopsychosocial lifestyle factors in CGH patients was investigated. It was emphasized that modifiable factors like poor sleep quality and depression are associated with more frequent headaches and a higher likelihood of chronic CGH. These findings suggest that CGH should be considered within the framework of a biopsychosocial model. Although poor sleep and depression are common features of chronic pain in general, addressing these factors in CGH may still help reduce the risk of chronification.

Another factor influencing postural alignment is BMI, which has biomechanical implications. In this study, a significant negative correlation was found between BMI and CVA, and it was determined that an increase in BMI is an independent predictor of CVA decrease. In the literature, it has been reported that an increase in BMI alters cervical sagittal alignment, increasing the sagittal vertical axis and causing a forward shift in cervical posture [35]. This change may increase the mechanical load on the cervical spine, leading to a reduction in lordosis. Indeed, patients with a higher BMI were found to have reduced C2–C7 cervical lordosis, and this difference became more pronounced during follow-up [35]. Furthermore, high BMI was shown to increase the risk of adjacent segment degeneration, which may contribute to cervical postural instability and increased musculoskeletal load, ultimately leading to postural dysfunction [35]. Therefore, weight management in CGH patients can be considered together with approaches to correct postural dysfunction.

Finally, the therapeutic implications of postural correction are worth emphasizing. It has been shown in the literature that exercise programs focusing on the correction of postural disorders and maintaining muscle balance are effective in the treatment of CGH. Nobari M *et al*. [36] reported that corrective exercises in individuals with FHP significantly reduced headache severity, duration, and frequency, while also improving the neck disability index. Similarly, in another study, it was stated that postural arrangements and exercises positively affected the mechanical properties of the upper cervical muscles and contributed to the relief of headache symptoms, with improvements in the craniovertebral angle [37]. These findings in the literature align with our results regarding the effects of FHP on CGH symptoms. For this reason, it is recommended to apply specific exercise programs in order to correct postural disorders and maintain muscle balance in the treatment of CGH.

This study has some limitations worth acknowledging. First, the absence of a control group limits the ability to compare differences between individuals with CGH and healthy individuals. Therefore, controlled studies are needed to understand better the relationship between CGH and clinical and environmental factors. Second, the observational and retrospective design of our study limits the ability to draw definitive causal inferences, which would ideally require an experimental approach such as a randomized controlled trial. As this study has a cross-sectional design, no causal relationship can be established between forward head posture and cervicogenic headache. In particular, prospective research is needed to understand better the changes in the effects of parameters such as BMI, CVA, and sleep quality on CGH over time. Third, the generalizability of the findings is limited since the participants were selected only from patients who applied to our outpatient clinic with complaints of neck pain. Finally, the risk of type I error due to multiple comparisons should be acknowledged, and future studies using correction methods are recommended to confirm these findings.

## 5. Conclusions

In this study, important findings suggesting that postural changes may be related to CGH have been obtained. In particular, it was determined that decreased CVA was an independent predictor of CGH, while BMI and pain severity were associated with postural changes. It is thought that FHP may be associated with CGH, possibly through increased biomechanical load on the cervical spine. In this context, clinical implementation of posture-corrective exercises and structured weight management programs may serve as supportive strategies to improve biomechanical balance and reduce CGH symptoms in daily practice. In addition, poor sleep quality was found to be an independent predictor of CGH. These results suggest that CGH may be shaped not only by biomechanical factors but also by biopsychosocial factors. Therefore, multidisciplinary management approaches that integrate physiotherapy, psychological support, and lifestyle interventions could be more effective in the clinical care of CGH. Furthermore, future prospective and randomized controlled trials are needed to evaluate the long-term effects of postural rehabilitation, sleep quality enhancement, and weight control interventions on CGH incidence and symptom severity.
